# Molecular identification of the source of an uncommon tularaemia outbreak, Germany, autumn 2016

**DOI:** 10.2807/1560-7917.ES.2019.24.18.1800419

**Published:** 2019-05-02

**Authors:** Daniela Jacob, Kristin Köppen, Aleksandar Radonić, Berit Haldemann, Philipp Zanger, Klaus Heuner, Roland Grunow

**Affiliations:** 1 Highly Pathogenic Microorganisms (ZBS 2), Centre for Biological Threats and Special Pathogens, Robert Koch Institute, Berlin, Germany; 2 These authors contributed equally to this work; 3 Cellular Interactions of Bacterial Pathogens, ZBS 2, Robert Koch Institute, Berlin, Germany; 4 Genome Sequencing (MF 2), Methodology and Research Infrastructure, Robert Koch Institute, Berlin, Germany; 5 Bioinformatics (MF 1), Methodology and Research Infrastructure, Robert Koch Institute, Berlin, Germany; 6 Federal State Agency for Consumer & Health Protection Rhineland-Palatinate, Koblenz, Germany; 7 Department of Infectious Diseases, Medical Microbiology and Hygiene, University Hospitals, Heidelberg, Germany; 8 Heidelberg Institute of Global Health, Unit of Epidemiology and Biostatistics, University Hospitals, Heidelberg, Germany

**Keywords:** tularaemia, Francisella, Francisella tularensis, outbreak, next generation sequencing, transmission, Apodemus sylvaticus, grape, grape harvest, must, wine

## Abstract

**Background:**

In 2016, an uncommon outbreak of oropharyngeal tularaemia involving six human cases occurred in Germany, caused by drinking contaminated fresh must after a grape harvest.

**Aim:**

We describe the details of laboratory investigations leading to identification of the outbreak strain, its characterisation by next generation sequencing (NGS) and the finding of the possible source of contamination.

**Methods:**

We incubated wine samples in different media and on agar plates. NGS was performed on DNA isolated from young wine, sweet reserve and an outbreak case’s lymph node. A draft genome of the outbreak strain was generated. Vertebrate-specific PCRs using primers targeting the mitochondrial cytochrome b gene and product analyses by blast search were used to identify the putative source of must contamination.

**Results:**

No bacterial isolate could be obtained. Analysis of the draft genome sequence obtained from the sweet reserve attributed this sequence to *Francisella tularensis* subsp. *holarctica*, belonging to the B.12/B.34 phylogenetic clade (erythromycin-resistant biovar II). In addition, the DNA sequence obtained from the case’s isolate supported our hypothesis that infection was caused by drinking contaminated must. The vertebrate-specific cytochrome b sequence derived from the young wine and the sweet reserve could be assigned to *Apodemus sylvaticus* (wood mouse), suggesting that a wood mouse infected with *F. tularensis* may have contaminated the must.

**Conclusion:**

The discovered source of infection and the transmission scenario of *F. tularensis* in this outbreak have not been observed previously and suggest the need for additional hygienic precautionary measures when processing and consuming freshly pressed must.

## Introduction

*Francisella tularensis*, a facultative intracellular Gram-negative bacterium, is the causative agent of tularaemia, a zoonotic disease. Outbreaks in humans are often associated with exposure to infected animals, contaminated water or aerosols, and different arthropod vectors [1-5]. The clinical manifestation mainly depends on the route of infection, and the two main subspecies—*F. tularensis* subsp. *tularensis* and subsp. *holarctica*—are clinically relevant [1,6].

In Germany, *F. tularensis* is endemic [7-14] and 20–40 cases of tularaemia are reported per year, with numbers increasing since 2005, indicating that tularaemia is a rare but re-emerging disease [7]. The only *Francisella* subspecies known to cause tularaemia in Germany is *F. tularensis* subsp. *holarctica* (*Fth*). In addition, a further *Francisella* species (*Francisella* sp. strain W12–1067, environmental isolate) was identified in Germany, but it is not yet known whether this species is pathogenic for humans [15].

On 2 October 2016, there was an unusual outbreak of oropharyngeal tularaemia involving six cases in a group of 29 persons attending a grape harvest in Rhineland-Palatine, Germany [16]. Grapes collected by a mechanical harvester were pressed at the winery and participants had the opportunity to consume the fresh must at the end of the harvest. Because tularaemia was not initially considered as a possible differential diagnosis, a delay of about 5 weeks occurred in confirming the diagnosis of tularaemia. One of the six serologically confirmed tularaemia cases had complicated tularaemia and was hospitalised with pharyngitis and cervical abscess-forming lymphadenopathy.

In order to identify and characterise the causative agent of the outbreak, we analysed the contaminated must-derived products, sweet reserve (SR) and young wine (YW), as the contaminated must was no longer available for investigation. The SR is fumigated must that already contains low amounts of ethanol and the YW is must with added yeasts to start the fermentation process. In addition, we used lymph node material (PL) from the patient with complicated tularaemia for further investigation [16].

The aim of this report was to describe details of the laboratory investigations leading to the finding of the likely source of contamination of the must.

Moreover, by using next generation sequencing (NGS), we further characterised the outbreak strain and confirmed the presence of its DNA in the clinical material of one of the patients.

## Methods

### DNA extraction

We obtained an aspirate lymph node sample from one of the six patients of the must-associated outbreak who had a protracted clinical course with abscess-forming lymphadenitis (Wetzstein N, Wolf T, personal communication, December 2016). The PL sample was collected directly after suspicion of the outbreak, i.e. 6 weeks after the event leading to the outbreak, which had not been recognised earlier. The contaminated must-derived products (sort 1A [16]), sweet reserve (RKI -sample number SR; A-856/3 (from sort 1A) and young wine (YW; A-856/2; from sort 1A), were collected by public authorities 3 weeks after the outbreak and were investigated. An additional YW (A-856/1; from sort 1B, pressed directly after sort 1A [16]) was also collected and studied. A total of 200 mL of each specimen were concentrated by centrifugation (45 min, 4,500 × *g*). Each pellet (SR, very thin pellet; YW, more pellet material, mainly yeast cells) was resuspended in 2 mL supernatant and further centrifuged at 20,000 × *g* for 5 minutes. Each pellet was again resuspended in 100 µL. DNA extractions were performed from the resuspended pellets (100 µL) and from 100 µL of aspirate (lymph node fluid) from the patient’s left neck lymph node (PL; A-877/1), according to the protocol for Gram-negative bacteria in the manufacturer’s instructions, using either the MoBio Power Soil DNA isolation kit (MoBio Laboratories, Carlsbad, California (CA), United States (US)) or the Qiagen DNeasy Blood and Tissue kit (Qiagen, Hilden, Germany), respectively. During the extraction, at the point of the proteinase K digestion, a plasmid with an artificial sequence insert was added to each sample as an internal extraction and amplification control (pKoMa2 [17]). Elution of DNA was performed twice with 75 µL (SR, YW) and 50 µL (PL) of Aqua bidest, respectively.

### Singleplex and multiplex real-time PCRs, RD1-PCR

Multiplex real-time PCR (5′ nuclease assay, TaqMan technology) targeting *fopA* and *tul4* specific for *F. tularensis*, in combination with the extraction and amplification control targeting KoMa2, were performed with oligonucleotides and probes as described in Table 1. A singleplex real-time PCR assay was performed from the clinical human sample for the detection of *c-myc* as a process control (oligonucleotide and probe; Table 1). Both real-time PCR assays were run in a total volume of 25 µL, including 5 µL of DNA of the samples to be analysed. Samples were analysed in duplicate in each run. The reaction mix components were 6.25 µL TaqMan Environmental MasterMix 2.0 (ThermoFisher, Henningsdorf, Germany), 10 pmol/µL primers (0.75 µL each) and probes (0.25 µL each). Amplification was performed in an Applied Biosystems 7500 Real-Time PCR System (ThermoFisher Scientific, Langenselbold, Germany), each run with an initial denaturation step at 95 °C for 10 minutes, followed by 40 cycles containing a denaturation step at 95 °C for 15 seconds and a combined primer annealing and elongation step at 60 °C for 60 seconds.

**Table 1:** Oligonucleotides used for PCR reactions, tularaemia outbreak, Germany, autumn 2016

PCR primers and probes	Sequence (5´–3´)	GenBank number	Position	PCR product (bp)	Ref
***F. tularensis* multiplex PCR**					
Ft-fopA-F	TTGGGCAAATCTAGCAGGTCA	M93695.1	821–841	101	[2]
Ft-fopA-R	ATCTGTAGTCAACACTTGCTTGAACA	M93695.1	921–896		
Ft-fopA-TM-Multi	FAM- AAGACCACCACCAACATCCCAAGCA-BHQ-1	M93695.1	875–851		
Ft-tul4-F	AGATTACAATGGCAGGCTCC	M32059.1	256–275	144	
Ft-tul4-R	AGCTGTCCACTTACCGCTACA	M32059.1	399–379		
Ft-tul4-TM-Multi	Cy5- TTCTAAGTGCCATGATACAAGCTTCCCAA-BHQ-2	M32059.1	282–310		
KoMa2-For	GGTGATGCCGCATTATTACTAGG	ND^a^	198–220	139	[17]
KoMa2-Rev	GGTATTAGCAGTCGCAGGCTT		336–316		
KoMa2-TM	JOE-TTCTTGCTTGAGGATCTGTCGTGGATCG-BHQ-1		224–251		
***c-myc* singleplex PCR**					
c-myc hum for	GCC AGA GGA GGA ACG AGC T	NG_007161.2	10296–10314	81	This study
c-myc hum rev	GGG CCT TTT CAT TGT TTT CCA	NG_007161.2	10376–10356		
c-myc human TM	FAM-TGC CCT GCG TGA CCA GAT CC-TAMRA	NG_007161.2	10330–10349		
**Region of difference PCR**					
RD1/F	TTT ATA TAG GTA AAT GTT TTA CCT GTA CCA	AF469614	1–30	Variable, dependent on subspecies	[18]
RD1/R	GCC GAG TTT GAT GCT GAA AA	AF469614	15221503		
**Cytochrome b gene PCR**					
UNFOR403	TGAGGACAAATATCATTCTGAGG	AB033695.1	403–425	623	[29]
UNREV1025	GGTTGTCCTCCAATTCATGTTA	AB033695.1	1025–1004		

ND: not deposited; Ref: reference.

^a^ The synthetic sequence (5′–3′) of the internal control KoMa2 is as follows: CGGCTCTAGCGCTGGTGGAGGTTAGAGTTCTCTGACATACGTGCTTCTGAACGGTAGGGAGTTGACGGACTGAGGGTAGGAGTGCTTAGCGTAGGAGTATTAGGTGGCGTGCTGTGGTGGTCGCGTGTGGGTTTAAGGAGTAAGTGCATTGAGGATTCAGGTGACACAGTAACGTGCAGTTGACTGGTGATGCCGCATTATTACTAGGCGATTCTTGCTTGAGGATCTGTCGTGGATCGGGGAGCGCAAACCTTACATGATATGTCTAAAATAGCTTTATGCCCTGCGATCGACCATATTTAAAAGCCTGCGACTGCTAATACCTATAGACTGAGGAGGGATTGAGAGAGCGAAAAATAGAGCAGACTGTATGATTACTATCGCGTGCCATCTCTAACTTTGCATAAGCGTCGTATTATTGGCAGCTACGAGTATCACGATTAGTCCGAACTAGTGGC (the underlined nucleotides denote the priming sites for the primers KoMa2-For and KoMa2-Rev and the probe KoMa-TM).

The calculation of genome equivalents for *tul4* and *c-myc* was done for the SR, YW and PL by using the plasmids of the TOPO TA vector cloning kit (Invitrogen, Karlsruhe, Germany), containing the respective target region for *tul4* of *F. tularensis* or *c-myc* as quantitative standards. For this purpose, in each real-time PCR run, standard control plasmids at the concentration of 10^2^, 10^4^ and 10^6^ copies/25 µL for the different targets (*tul4*, *fopA* and *c-myc*) were added to generate target-specific standard curves that allow the calculation of the quantity of samples.

### PCR for *Francisella* *tularensis* subspecies differentiation

The block PCR of the region of difference 1 (RD1-PCR) was used for the subspecies differentiation of *F. tularensis*. The PCR was carried out using the DreamTaq Polymerase (ThermoFisher, Hennigsdorf, Germany) with 15–100 ng of template DNA, according to the protocol described by Broekhuijsen et al. [18].

### Next generation sequencing

For the sequencing of the YW and SR samples (both of sort 1A) and PL, Illumina sequencing in combination with Nextera XT library generation was used (Illumina, San Diego, CA, US). DNA was quantified by using the Qubit dsDNA HS Assay Kit (Life Technologies, Darmstadt, Germany). Library generation was done with the Nextera XT DNA Sample Preparation Kit (Illumina, San Diego, CA, US), following the manufacturer’s instructions. The library normalisation step was skipped. Libraries were quantified by using the KAPA Library Quantification Kit for Illumina (Kapa Biosystems, Wilmington, Massachusetts (MA), US) and were pooled before sequencing. Library size was determined by using the High Sensitivity DNA Analysis Kits for the 2100 Bioanalyzer Instrument (Agilent Technologies, Waldbronn, Germany). The library pool was sequenced on a MiSeq instrument (Illumina, San Diego, CA, US). For cluster generation and sequencing, the MiSeq Reagent Kit v3 600 cycles was used to sequence 300 + 300 bases in paired-end mode.

### Mapping and generation of consensus sequence

Sequence quality assessment and trimming was performed with our quality control pipeline QCumber, developed in house [19], which combines the external tools Trimmomatic [20] and FastQC [21]. Furthermore, the pipeline uses the tools Bowtie2 [22] and Kraken [23] to analyse the origin of sequenced reads. Results of the taxonomic classification performed by Kraken on a Kraken-customised database consisting of bacterial, archaeal, viral and fungal genomes were visualised with Krona [24].

Trimmed reads were then mapped to the reference genomes of the expected background organisms (for SR and YW: yeast genome (GCA_000146045.2) and grape genome (GCA_000003745.2); for the patient sample: human genome (GRCh38) using Bowtie2). Reads not mapping to the background organisms’ genomes were then mapped to the *Fth* live vaccine strain (LVS) (NC_007880.1) genome and a consensus sequence (draft genome sequence) was generated using Geneious (version R9.1.3 [25]; with a threshold of 75% and following the IUPAC code for ambiguities). The draft genome sequence generated from DNA isolated from SR (*Fth*-Must) has been submitted to GenBank (CP024807) and raw reads have been uploaded to the Sequence Read Archive (SRA, https://www.ncbi.nlm.nih.gov/sra; BioProject PRJNA417909).

### Phylogenetic analysis

The draft genome sequence of *Fth*-Must was aligned with eight *Fth* reference genomes (OSU18, FTNF002–00, FDC409, FSC162, FDC407, FSC200, LVS and FDC408) and four draft genomes of *Fth* isolates (A-635, Fth-07, A-810/1 and A-663), representing main clades [26,27] using progressive Mauve alignment (MUSCLE 3.6). The phylogenetic tree was constructed by Geneious (Geneious 10.0.5) using the neighbour-joining method (Tamura–Nei, outgroup: OSU18; 100 bootstrap replicates). The canSNP analysis was performed using published canSNP positions [26,28] to confirm the clade and subclade determination of the draft genome sequence of *Fth*-Must. Sequences used are given in Table 2.

**Table 2:** canSNP analysis of the nucleotide sequences of *Fth*-Must and *Fth*-Patient, tularaemia outbreak, Germany, autumn 2016

Clade or SNP	Ancestral	Derived	LVS	Must	Patient
**Clade**					
B.4	AAATCCtGCAGCAAA	AAATCCaGCAGCAAA	A	A	NA
B.5	GGCACAAGcTTTAGCTGA	GGCACAAGtTTTAGCTGA	D	D	NA
B.6	CCCTGCTAcAGAATCAT	CCCTGCTAtAGAATCAT	A	A	NA
B.12	GTCAATATAtCGAAAATGGT	GTCAATATAaCGAAAATGGT	D	D	D (1 read)
B.72	CTCAGTAGAgGTGATTTC	CTCAGTAGAtGTGATTTC	D	D	NA
B.71	GTTTTCACAgCAAAATGCC	GTTTTCACAaCAAAATGCC	A	A	NA
B.13	GGCGAATCtCTAGATGAT	GGCGAATCcCTAGATGAT	D	D	NA
B.39	CTTCAACTGgCTGACCT	CTTCAACTGaCTGACCT	A	A	NA
B.26	GTTGCCgATTGTCACT	GTTGCCtATTGTCACT	D	D	NA
B.43	ATCTAGTGcTTGTCTCA	ATCTAGTGaTTGTCTCA	A	A	NA
B.23	CGCCTCTAAgAGTATCTT	CGCCTCTAAtAGTATCTT	D	A	NA
B.42	GGTTGAATgTATGCAA	GGTTGAATtTATGCAA	A	D	NA
B.21	TATAATATGcGTAGCTGC	TATAATATGtGTAGCTGC	A	A	NA
B.33	CGCCAAAAgCACTACTT	CGCCAAAAaCACTACTT	A	D	(2 reads)^a^
B.34	GCTGGATcTAGAGAAG	GCTGGATtTAGAGAAG	A	D	NA
B.75	CTCTTAGCgCTAAAAACCG	CTCTTAGCaCTAAAAACCG	A	A	NA
**SNPs**					
rrl.1^b^	AATGACCGATAGTGaACTAGTACCGTGAG	AATGACCGATAGTGgACTAGTACCGTGAG	D	D	NA
rrl.2^b^	CCCGCGGTTAGACGGAaAGACCCCGTGAA	CCCGCGGTTAGACGGAcAGACCCCGTGAA (3 reads)	D	D	(3 reads)

A: ancetral; D: derived; Fth: *F. tularensis* subsp. *holartica*; LVS: live vaccine strain; NA: no reads available; SNP: single nucleotide polymorphism.

^a^ Two SNPs (C → T; LVS position bp 78,630, FTL_0082 and A → G, LVS position bp 1,011,166, FTL_1056) were only found in eight sequenced *Fth* B.33 strains.

^b^ B.12 is associated with erythromycin resistance; in addition, rrl.1and rrl.2 SNPs in the 23S rRNA gene *rrl* (A453G and A2059C, respectively; *Escherichia coli* numbering) are directly associated with the erythromycin resistance of *Fth* strains of clade B.12 [30].

### PCR detecting vertebrate cytochrome b and DNA cloning

A vertebrate-specific PCR assay was used to identify mammalian DNA within all SR and YW samples. As described by Kent and Norris, the primers UNFOR403 and UNREV1025 (Table 1) specifically detect the mammalian mitochondrial cytochrome b gene [29]. Therefore, a PCR was performed using the TopTaq DNA polymerase (Qiagen, Hilden, Germany), according to manufacturer’s instructions (for each PCR reaction (50 µL): 5 µL of 10x TopTaq PCR Buffer, 1 µL of dNTPs (Sigma Aldrich, St. Louis, Missouri, US), 10 µL of 5x Q-Solution, 1 µM UNFOR403, 1 µM UNREV1025, 0.5 µL of TopTaq DNA Polymerase and 22.5 µL of RNase-free water). Ten µL of the preprocessed DNA samples was used as a DNA template in each PCR reaction. As a positive control, 2 µL of sheep blood was directly pipetted into the PCR reaction. Chromosomal DNA of *Francisella* sp. strain W12–1067 and water served as negative controls. PCR amplification was done using a Thermocycler TRIO-Thermoblock (Biometra, Göttingen, Germany) involving initial denaturation (3 minutes, 94 °C), then 35 cycles including a denaturation step (30 seconds, 94 °C), an annealing step (30 seconds, 57 °C) and an extension step (1 minute, 72 °C), followed by a final extension (10 minutes, 72 °C). Subsequently, PCR products were separated according to their size in a gel electrophoresis. The expected PCR fragment (623 bp) was extracted using Wizard SV Gel and PCR Clean-Up System (Promega, Madison, Wisconsin (WI), US).

The isolated DNA was then cloned into a vector for subsequent sequencing of the insert DNA. For the cloning of the UNFOR403–UNREV1025 PCR product, the pGEM-T Easy Vector system was used according to the manufacturer’s instructions (Promega, Madison, WI, US). Briefly, 5 µL of gel-purified PCR product was ligated overnight into pGEM T Easy vector using DNA T4 ligase. Next, 2 µL of the ligation reaction was transformed into chemical competent *Escherichia coli* cells (Top10 cells: ThermoFisher, Waltham, MA, US). Recombinant *E. coli* cells were selected by growing on Luria-Bertani (LB) agar plates containing 100 µg/mL ampicillin, 0.1 mM IPTG and 0.006% X-Gal. White clones were tested in PCR using the primer combination UNFOR403 and UNREV1025. Insert DNA of all clones was sequenced and a BLAST analysis was performed.

## Results

### Analyses of must products and patient samples

**Figure 1:**
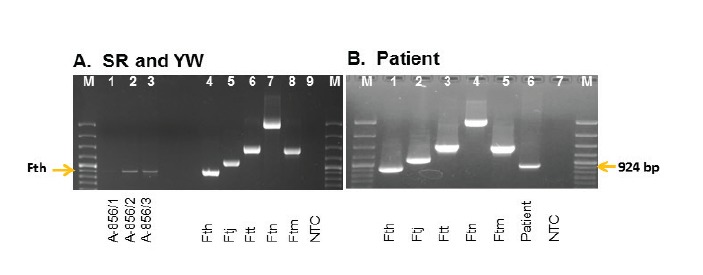
Amplification fragments by RD1-PCR analyses of the sweet reserve, young wine and patient sample, tularaemia outbreak, Germany, autumn 2016

We incubated samples of SR and YW in medium T, on cystein-heart-blood-agar (CHAB) plates and on CHAB agar plates containing selective antibiotics (CHAB-PACCV) [27], but we were not able to obtain a *Francisella* strain. *Fth* DNA was detected in samples of SR and YW by real-time PCR targeting *tul4* and *fopA.* The subspecies was identified and confirmed by RD1-PCR analysis in both YW and SR (Figure 1A, lanes 1–3), as well as in the PL aspirate sample (Figure 1B, lane 6). The obtained DNA sample was used for NGS analysis to identify the outbreak strain. The PL sample and the SR and YW samples (sort 1A and 1B) were analysed by quantitative PCR analysis and revealed the following genome equivalents per mL: SR, 1.0 x 10^2^ (sort 1A, sample A-856/3); YW, 1.7 x 10^4^ (sort 1A, sample A-856/2); YW 4.4 x 10^2^ (sort 1B, sample A-856/1)(data from [16]); and PL 1.7 x 10^6^ (A-877/1).

M: DNA marker; NTC: no template control; SR: sweet reserve; YW: young wine.

In panel A, sample 1 = A-856/1 YW sort 1B, 2 = A-856/2 YW sort 1A and 3 = A-856/3 SR sort 1A. In panel B, the patient sample = lane 6. In both panels, controls and fragment sizes of lane 4/1 = *Francicella tularensis* subsp. *holarctica* (Fth) 924 bp; 5/2 = *F. tularensis* subsp. *japonica* (Ftj) 1,135 bp; 6/3 = *F. tularensis* subsp. *tularensis* (Ftt) 1,522 bp; 7/4 = *F. tularensis* subsp. *novicida* (Ftn) 3,322 bp; 8/5 = *F. tularensis* subsp. *mediasiatica* (Ftm) 1,453 bp; 9/7 = NTC: no template control; M = DNA ladder: 100 bp GeneRuler.

This suggested that the SR and YW (sort 1A) DNA and PL-DNA contained enough *Francisella* DNA to be successfully sequenced by NGS. Since it is obvious that patient samples contain large amounts of human DNA, which could make it difficult to receive a conclusive result in NGS sequencing, we checked the *tul4*/*c-myc* (*Francisella* DNA/human DNA) ratio of the patient sample DNA. *Tul4* is a *Francisella*-specific gene and *c-myc* encodes for a human transcription factor. The *tul4*/*c-myc* ratio was 1:15, indicating that there was enough *Francisella* DNA within the sample to be analysed by NGS sequencing.

### Next generation sequencing and identification of the outbreak strain DNA

**Figure 2:**
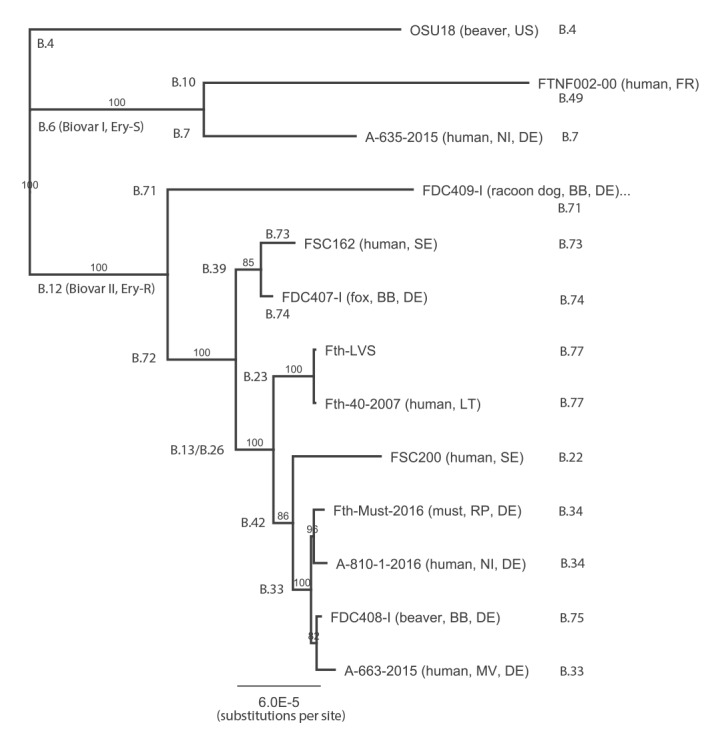
Neighbour-joining phylogenetic tree showing the relationship between different *Fth* strains and the *Fth*-Must DNA, tularaemia outbreak, Germany, autumn 2016

DNA from SR and YW (sort 1A) was used to perform NGS sequencing. After NGS, quality control and read trimming was performed. Taxonomic classification of trimmed reads (Krona plots, data not shown) revealed a high amount of *Saccharomyces cerevisae* (85%) reads, as well as plant- and soil-associated bacteria (*Gluconobacter, Pseudomonas syringae, Tatumella, Pantoea, Komagataeibacter*). To remove reads originating from background organisms, all reads (YW: 27,345,303; SR: 17,931,396) were mapped to the yeast genome (GCA_000146045.2). Subsequently, unmapped reads were mapped to the grape genome (GCA_000003745.2). The unmapped reads obtained (YW: 3,674,761 and SR: 17,709,501) were mapped to the genome of *Fth* strain LVS (NC_007880.1). Of the reads of YW and SR, 1.9% (71,201) and 9.6% (1,696,328) mapped to the *Francisella* genome. However, there was still a high number of unmapped and unclassified reads, mainly in the SR sample. The results demonstrate that both the YW and the SR were contaminated with *Francisella* DNA. Reads from the SR mapped to the *Fth* LVS genome (1,895,994 bp) were used to generate a consensus sequence of *Fth*-SR (*Fth*-Must). The approximately 1.7 x 10^6^ mapped reads are distributed over the whole genome of *Fth* LVS (1,696,328 reads 50–238 bp in length, mean: 187 ± 53 bp; mean coverage: 167) providing a first draft *Fth* genome sequence (*Fth*-Must, 1,895,952 bp). This DNA sequence was aligned with whole genome sequences of different *Fth* strains and was used to generate a phylogenetic tree (Figure 2). The *Fth*-Must DNA was found to cluster with another human *Fth* isolate (A810–1) from Germany in the phylogenetic subclade B.34 (Figure 2). In addition, we performed an in silico analysis of the canSNP analysis scheme, confirming that isolate *Fth*-Must corresponded to the B.12 clade (erythromycin-resistant strains) and to subclade B.34 (Table 2). The affiliation to the B.12 clade was further confirmed by the identification of two SNPs within the 23S rRNA gene *rrl* that were recently found to be specific for strains belonging to this clade [30].

The alignment was performed using progressive Mauve alignment (MUSCLE 3.6) and the phylogenetic tree was built with Geneious (Geneious 10.0.5) (Tamura–Nei, Neighbour-Joining, 100 bootstrap replicates), setting *Fth* OSU18 strain as outgroup. The branch labels indicate the posterior probability and the branch length corresponds to the evolutionary distance (substitutions per site). For details of canSNP analysis B.X clusters and subclusters, see Table 2 and references [26-28]. German federal states: Brandenburg (BB); Mecklenburg-Western Pomerania (MV); Lower Saxony (NI); Rhineland-Palatinate (RP). Countries: France (FR), Germany (DE), Lithuania (LT), and Sweden (SE).

In addition, NGS sequencing of the PL-DNA generated 22,116,655 reads and, as expected, 98.6% of the reads mapped to the human genome sequence (GRCh38). However, 691 and 690 reads (covering ca 107,073 bp of the whole *Fth* LVS genome sequence) mapped to the genome of *Fth* LVS and *Fth*-Must, respectively. We analysed the obtained reads for the presence of canSNPs used for subtyping and found one read mapping to the B.12 canSNP and two reads mapping to two respective SNPs only found in strains of subclade B.33 (Table 2). In addition, three reads mapped to two of three copies (present at the genome of *Francisella*) of SNP rrl-2 in the *rrl* gene (Table 2). The results confirmed that the isolated *Francisella* DNA belonged to a *Francisella* strain of clade B.12 associated with erythromycin resistance [30] and at least to sub-clade B.33 (Figure 2), although the low coverage did not allow a clear identification. Further analyses could not be performed due to the restricted amount of DNA. Altogether, the results suggested that this patient was infected with the same *Fth* strain that was identified in the SR and served as must during the grape harvest. Tularaemia was also confirmed for all other diseased participants of the grape harvest [16].

### Identification of the putative contamination source

Despite the identification of the outbreak strain DNA, the question of how the must had been contaminated with *Francisella* was still not answered. Since small rodents are occasionally found in mechanically harvested grapes [16], we used the unmapped reads of the last mapping step (using DNA from the SR, see above) and performed an additional mapping to a mouse genome (GCA_000001635.7). About 2,600 reads were found mapping to this mouse genome, but a definite identification of the species was not successful. However, based on the obtained results, we analysed the samples for the presence of vertebrate DNA to identify the species that may have contaminated the must with infectious *Francisella*.

**Figure 3:**
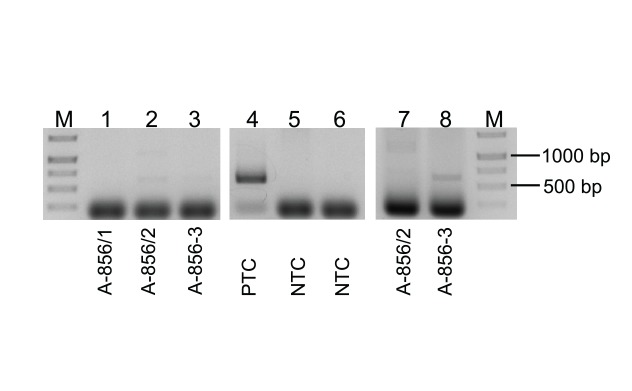
Detection of mammalian DNA in must samples, tularaemia outbreak, Germany, autumn 2016

For this purpose, we used a vertebrate-specific primer pair to amplify specifically the mitochondrial cytochrome b gene if present in the must. The different sample DNAs (A-856/1–3) were used in the PCR reaction. A PCR band of the expected size (623 bp) could be detected in YW and SR samples of sort 1A (Figure 3, lanes 2/3 and 7/8), but not in the YW of sort 1B (Figure 3, lane 1). The amplified DNA was isolated from the agarose gel, pooled and cloned into vector pGEM Teasy. The insert DNA was checked for specificity by PCR analysis (data not shown). The cloned insert DNA of all 10 clones tested could be amplified and the insert DNA of all clones was sequenced. A first basic local alignment search tool (BLAST) analysis identified all 10 PCR products analysed as the cytochrome b gene of *Apodemus*.

M: DNA marker; NTC: negative template control; PTC: positive template control; SR: sweet reserve; YW: young wine.

Samples A-856/1–3 (lanes 1–3 and lanes 7–8) were analysed in PCR with primers detecting vertebrate cytochrome b (UNFOR403, UNREV1025, fragment size 623 bp). As a positive template control, sheep blood (PTC, lane 4) was used. Chromosomal DNA of *Francisella* sp. strain W12–1067 (NTC, lane 5) and water (NTC, lane 6) served as negative template controls. DNA ladder (M): GeneRuler 1 kb DNA Ladder. Sample A-856/1 = YW sort 1B, A-856/2 = YW sort 1A, A-856–3 = SR sort 1A.

**Figure 4:**
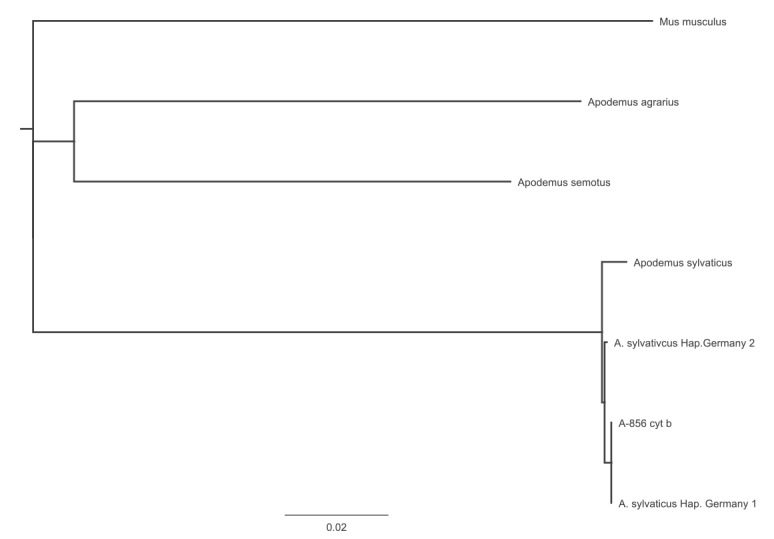
Neighbour-joining phylogenetic tree demonstrating the identified *cytochrome b* sequence belonging to *Apodemus sylvaticus*, tularaemia outbreak, Germany, autumn 2016

The alignment of the obtained cytochrome b consensus sequence of 578bp (cytochrome b PCR DNA product without primer sequences) with cytochrome b genes of different *Apodemus* species revealed that the obtained DNA sequence was 99.8% identical (one R at position 279) to the cytochrome b gene of a wood mouse (also called long-tailed field mouse) (*Apodemus sylvaticus* (Haplotype Germany-1/Haplotype France)). The phylogenetic tree of this alignment is shown in Figure 4. The DNA region of the cytochrome b gene of haplotype Germany-1 and -2 exhibited only one SNP at position 329 (Germany-1: T, Germany-2: C). These results suggested that a wood mouse infected by *Fth* might have been the contamination source of the must consumed by participants in the grape harvest.

The alignment was performed using progressive Mauve alignment (MUSCLE 3.6) and the phylogenetic tree was built with Geneious (Geneious 10.0.5) (Tamura–Nei, neighbour-joining, 100 bootstrap replicates), setting *Mus musculus* as the outgroup. The branch length corresponds to the evolutionary distance (substitutions per site). cyt b: cytochrome b gene; Hap.: haplotype.

## Discussion

Freshly pressed must was served to some of the 29 participants in the grape harvest [16]. Wine yeast was added to a large portion of the must for the production of young wine and a smaller portion was fumigated and served as sweet reserve. In the SR and YW of sort 1A, *Francisella* DNA was detected at a high concentration and was confirmed to be *Fth* specific by PCR analysis (Figure 1). In sort 1B, pressed directly after sort 1A, only 440 genome equivalents per mL of *F. tularensis* was detected, suggesting a cross-contamination of this wine in the winepress [16].

NGS sequencing of DNA isolated from the YW (sort 1A) and SR revealed, as expected, more reads mapping to the yeast genome in the YW (8.6%) than in the SR (0.5%). In both unmapped reads, an equal but small proportion (ca 1%) of reads was mapped to the grape genome, suggesting that there was a low amount of grape DNA in the YW and SR. In addition, in the obtained unmapped reads, 1.9% and 9.6% of the YW and SR reads mapped to the *Fth* LVS genome, respectively, demonstrating that there was still a lot of *Francisella* DNA present in these samples. Half of the unmapped reads from the SR did not map to any known sequences in the Kraken-customised database, and some reads mapped to different plant- and soil-associated bacteria, probably environmental and grape-associated bacteria.

After NGS sequencing of DNA from the SR, we were able to generate a consensus sequence (draft genome, *Fth*-Must) covering nearly the whole genome of *Fth* LVS. This draft genome may contain regions shared between different organisms in the sample. However, the distribution of mapped NGS reads, as well as the phylogenetic tree and canSNP analysis, demonstrated a good quality of the generated consensus sequence. The phylogenetic analysis of the *Fth*-Must DNA sequence revealed that the DNA belonged to a strain that clustered into subclade B.34 (Figure 2). Although the obtained reads of the NGS analysis of PL-DNA covered only a small part of the *Fth*-Must sequence, the results confirmed the hypothesis that the patient from the outbreak had been infected by drinking must. Further, in 2016, we identified a *Francisella* isolate (A-810/1, data not shown) from a patient in Lower Saxony who had contracted tularaemia, confirming the presence of the subclade B.34 in Germany (Figure 2).

To identify the source that contaminated the must with *Fth*, we performed a vertebrate-specific cytochrome b gene PCR, a conserved mitochondrial gene used for phylogenetic investigations [31]. Sequencing of the obtained PCR products revealed a nucleotide sequence that was 99.8% identical to the cytochrome b gene of *A. sylvaticus* haplotype Germany-1/France, a wood mouse. *A. sylvaticus* is known to consume fruit, and it has been reported that this species can be infected with *Francisella* [32]. Different studies in Croatia, Germany, Hungary and Spain demonstrated that different small rodents, like *Myodes* (bank voles), *Sorex* (common shrew), *Microtus* (common vole), *Muscardinus* (common dormouse) and various species of *Apodemus* (*A. flavicollis*, *A. agrarius* and *A. sylvaticus*) can be infected by or can be carriers of *Francisella tularensis* [5,10,32-34]. The findings from these studies support our hypothesis of an infected wood mouse as the source that contaminated the must.

Unfortunately, the late suspicion of the tularaemia outbreak did not allow the isolation of the outbreak-causing strain from the must, the patient or the suspected mouse for further functional investigation. It should be emphasised that modern laboratory techniques made it possible that all genomic characterisation could be obtained from DNA only. More data on the occurrence of *F. tularensis* in the region, including in rodents and other wild animals, would be helpful for further risk assessment and greater awareness of tularaemia during wine production.

## Conclusion

Analysing this uncommon tularaemia outbreak, we were able to determine a draft genome sequence of the responsible *Francisella* strain, although no isolate could be obtained. Using this draft genome, a phylogenetic analysis was successful. Some reads exhibiting specific canSNPs identified in the DNA extracted from a patient’s lymph node supported the finding of our previous cohort study that the patients were infected by consuming the fresh must. In addition, through the identification of the putative source of the contamination, we could propose a most likely route of transmission for this outbreak: The automatic harvester may have collected a wood mouse (or its carcass) infected with a high dose of *Fth*, then transferred it to the mash car, contaminating the mash, the press and finally 730 L of must—an infectious dose for humans. Subsequently, this must was served to a group of participants in the grape collection and six people contracted tularaemia. Based on our results, it was suggested that additional hygienic precautions should be undertaken during wine harvesting and production. For example, rodent control should be put into practice throughout all steps of wine production and freshly pressed must for tasting should be produced from hand-picked instead of mechanically harvested wine grapes, since the latter is more difficult to control. As raw food products can be associated with a risk for infectious agents, pasteurisation before consumption is also recommended [35]. Further, our investigation shows that tularaemia should be considered when individuals fall ill with relevant symptoms after a grape harvest event.
